# The radiobiology of HPV-positive and HPV-negative head and neck squamous cell carcinoma

**DOI:** 10.1017/erm.2020.4

**Published:** 2020-07-02

**Authors:** Chumin Zhou, Jason L. Parsons

**Affiliations:** Cancer Research Centre, Department of Molecular and Clinical Cancer Medicine, University of Liverpool, 200 London Road, Liverpool L3 9TA, UK

**Keywords:** DNA damage, DNA repair, head and neck cancer, human papillomavirus, ionising radiation, radiation biology

## Abstract

Head and neck squamous cell carcinoma (HNSCC) is the sixth most common cancer worldwide, with reported incidences of ~800 000 cases each year. One of the critical determinants in patient response to radiotherapy, particularly for oropharyngeal cancers, is human papillomavirus (HPV) status where HPV-positive patients display improved survival rates and outcomes particularly because of increased responsiveness to radiotherapy. The increased radiosensitivity of HPV-positive HNSCC has been largely linked with defects in the signalling and repair of DNA double-strand breaks. Therefore, strategies to further radiosensitise HPV-positive HNSCC, but also radioresistant HPV-negative HNSCC, have focussed on targeting key DNA repair proteins including PARP, DNA-Pk, ATM and ATR. However, inhibitors against CHK1 and WEE1 involved in cell-cycle checkpoint activation have also been investigated as targets for radiosensitisation in HNSCC. These studies, largely conducted using established HNSCC cell lines in vitro, have demonstrated variability in the response dependent on the specific inhibitors and cell models utilised. However, promising results are evident targeting specifically PARP, DNA-Pk, ATR and CHK1 in synergising with radiation in HNSCC cell killing. Nevertheless, these preclinical studies require further expansion and investigation for translational opportunities for the effective treatment of HNSCC in combination with radiotherapy.

## Introduction

**Fig. 1.**
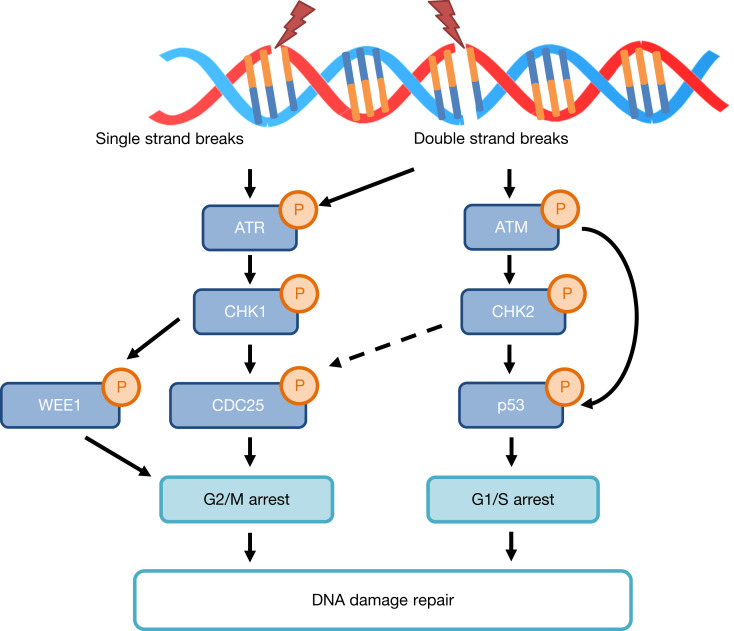
The DNA damage signalling pathway. Accumulation of DNA damage and replication stress leads to activation of ATM- and ATR-dependent signalling pathways mediated through the CHK2 and CHK1 effector kinases, respectively. Phosphorylation of the CDC25 phosphatases (leading to inactivation or ubiquitylation-dependent degradation) or phosphorylation-dependent stabilisation of the p53 tumour suppressor protein ultimately leads to cell-cycle arrest at either the G_2_M or G_1_S checkpoints, respectively allowing for DNA damage repair. WEE1 is also a target of CHK1 kinase activity, leading to G2/M checkpoint activation.

Head and neck cancer is the sixth most common cancer worldwide, with ~800 000 cases diagnosed every year (Ref. 1). The majority of these cancers are head and neck squamous cell carcinoma (HNSCC), and these arise mainly in the oropharynx, oral cavity, larynx and hypopharynx. The major risk factors for HNSCC are excessive tobacco and alcohol use, and the survival rate of HNSCC patients is ~40–50% with mortality mostly caused by regional recurrence and distant metastasis (Refs 2, 3). Therefore, HNSCC remains a significant public health concern. Human papillomavirus (HPV) type-16 infection is also a major risk factor related to 40–60% of oropharyngeal squamous cell carcinoma and ~40% of HNSCC combined (Refs 4, 5). Interestingly, HPV-positive patients display improved survival rates and prognosis in comparison with other HNSCCs (Refs 6, 7), which is linked with a better response to chemotherapy and radiotherapy (Ref. 8). This indicates that the two subtypes of oropharyngeal cancers harbour significantly varied clinicopathological and biological characteristics. At the biological level and on HPV infection, the viral oncogenes E6 and E7 cause ubiquitylation-dependent degradation of the tumour suppressor proteins p53 and retinoblastoma, which are involved in regulating cell-cycle progression and in coordinating DNA damage repair pathways (Ref. 9). This leads to lack of cell-cycle regulation at the G_1_S and G_2_M checkpoints, and subsequently promotes induction of genome instability, accumulation of chromosomal aberrations, tumour cell proliferation and ultimately in progression of the malignancy. In terms of the radiobiology of HNSCC, the critical cellular target for radiotherapy (ionising radiation; IR) is DNA, and the induction of DNA damage triggers the cellular DNA damage response (Fig. 1). The DNA damage activates the ataxia telangiectasia mutated (ATM) and ataxia telangiectasia and Rad3 related (ATR) serine–threonine protein kinases (Refs 10, 11). This subsequently causes the activation of effector kinases including checkpoint kinases 1 and 2 (CHK1 and CHK2) that activate cell-cycle arrest and promote DNA damage repair. In this review, we will summarise the impact of IR on DNA damage and the cellular pathways of DNA damage repair, but then focus on the proposed mechanisms contributing to the radiosensitivity of HPV-positive and HPV-negative HNSCC cells in vitro. We will also summarise the strategies that have been investigated to increase radiosensitivity of HNSCC in vitro and in vivo, with a particular focus on inhibitors targeting proteins involved in DNA damage repair and in cell-cycle checkpoint activation.

## DNA damage repair mechanisms

The major type of DNA damage induced by IR that contributes to cell killing is DNA double-strand breaks (DSBs). However, IR predominantly induces DNA base damage, including oxidised bases and sites of base loss (abasic sites), as well as DNA single-strand breaks (SSBs) that make up ~95% of the total IR-induced damage. Cells are equipped with several DNA damage repair mechanisms to combat this assault on DNA, and in particular DSBs are repaired by non-homologous end joining (NHEJ) and homologous recombination (HR) pathways that demonstrate cell-cycle dependence, whereas DNA base damage and SSBs are principally repaired by the base excision repair (BER) pathway.

### Base excision repair (BER)

**Fig. 2.**
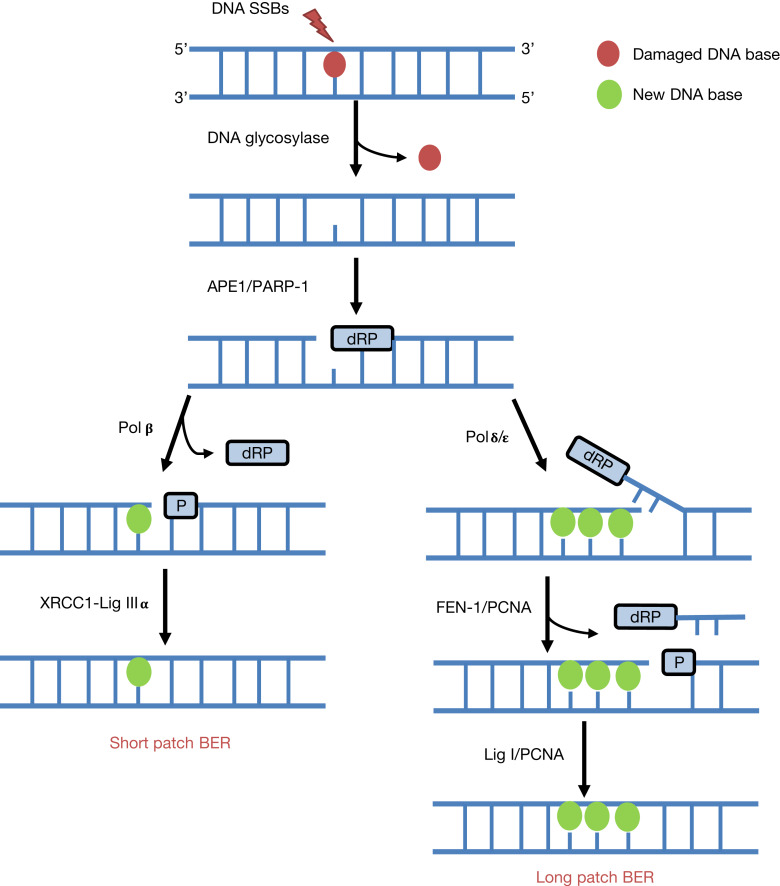
The BER pathway. BER is instigated by damage-specific DNA glycosylases that excise the damaged DNA base. Subsequently, APE1 promotes strand incision at the abasic site to generate a SSB containing a 5′-dRP end, which promotes PARP-1 binding. In short patch BER, single nucleotide incorporation and 5′-dRP removal is stimulated by Pol β, and DNA ligation by XRCC1-Lig IIIα. In contrast, in long-patch BER and following single nucleotide addition by Pol β, there is a polymerase switch to Pol δ/ε that insert 2–8 nucleotides and promote strand displacement. The flap structure is removed by FEN-1 with PCNA, and finally Lig I seals the SSB.

Given that mammalian cells encounter a significant amount of endogenous DNA base damage and SSBs because of reactive oxygen species generated during oxidative metabolism (>10 000 lesions per cell per day (Ref. 12)), as well as playing a key role in the repair of the majority of IR-induced DNA damage, BER is one of the most proactive pathways for DNA repair (Refs 13–15). BER is initiated by one of eleven damage specific DNA glycosylases that excise the damaged DNA bases (Refs 16, 17). However, the enzymes critical for responding to IR are 8-oxoguanine DNA glycosylase (OGG1), endonuclease III homologue (NTH1) and the endonuclease VIII-like proteins (NEIL1, NEIL2 and NEIL3) that predominantly remove oxidative DNA base damage (Fig. 2). Following DNA base damage excision, AP endonuclease 1 (APE1) is recruited to the resulting abasic site for strand incision generating a DNA SSB containing 5′-deoxyribosephosphate (dRP) and 3′-hydroxyl ends (Refs 18, 19). At this stage, poly(ADP-ribose) polymerase 1 (PARP-1) binds to the DNA intermediate because of its high affinity to the strand break (Refs 20, 21). DNA polymerase β (Pol β) then stimulates DNA synthesis by inserting the correct undamaged nucleotide into the repair gap and simultaneously removes the 5′-dRP group (Refs 22, 23). A complex consisting of X-ray cross-complementing protein 1 with DNA ligase IIIα (XRCC1-Lig IIIα) then seals the remaining nick in the DNA (Refs 24, 25). This is referred to as the short patch BER pathway, which is the predominant mechanism for repair of DNA base damage and SSBs (Ref. 26). In a small percentage of cases where the 5′-DNA ends are resistant to Pol β activity, then there is a polymerase switch to DNA polymerase δ/ε (Pol δ/ε) that initiate the long patch BER pathway by inserting a further ~2–8 nucleotides (Refs 27, 28). The flap structure containing the 5′-dRP group is excised by flap endonuclease 1 (FEN-1) in association with proliferating cell nuclear antigen (PCNA), and finally DNA ligase I (Lig I) is employed to seal the DNA ends.

### Non-homologous end joining (NHEJ)

**Fig. 3.**
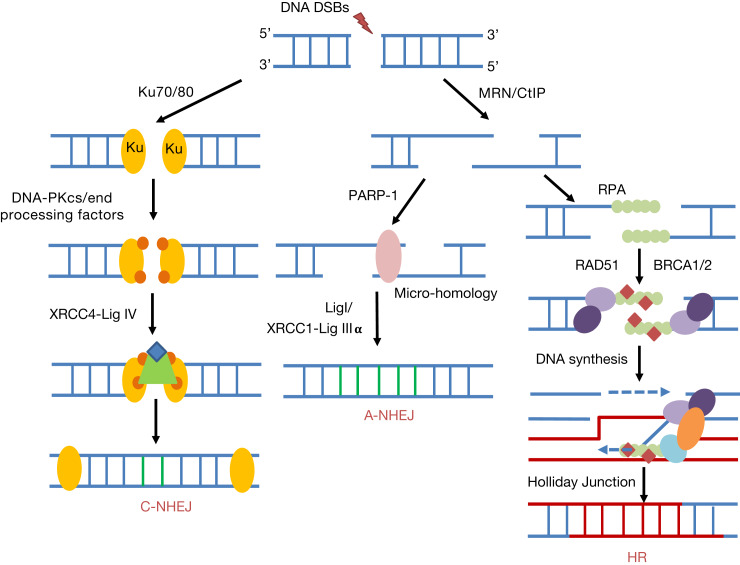
DNA DSB repair pathways. NHEJ can be divided into canonical NHEJ (C-NHEJ) and alternative NHEJ (A-NHEJ). C-NHEJ is stimulated by recruitment of the Ku70/80 heterodimer, DNA-PKcs and end-processing factors to the DSB ends. DNA ligation is promoted by XRCC4-Lig IV. In contrast for A-NHEJ, PARP-1 binds to the resected DNA ends generated by MRN and CtIP containing regions of micro-homology, and following DNA synthesis, ligation is performed by XRCC1-Lig IIIα or Lig I. During HR and following MRN/CtIP action in a BRCA1-dependent process, RPA binds to the single-stranded DNA regions generated. Further recruitment of BRCA2 and the RAD51 recombinase that displaces RPA to form a nucleoprotein filament promotes strand invasion, DNA synthesis and Holliday Junction formation and resolving.

NHEJ is the predominant repair pathway utilised for IR-induced DSBs throughout all phases of the cell cycle, and particularly in G_0_/G_1_ (Ref. 29). However, this is also an error-prone mechanism that can allow the addition or loss of nucleotides at the DSB ends. There are two well-defined sub-pathways of NHEJ (Fig. 3), namely canonical NHEJ (C-NHEJ) and alternative NHEJ (A-NHEJ) (Refs 30, 31). The first step of DSB recognition is the phosphorylation of the histone variant H2AX (γH2AX) stimulated by the protein kinases ATM and ATR (Ref. 32). In the C-NHEJ pathway, the DSB signalling protein 53BP1 is recruited to attach to the two DNA ends and interacts with the Ku70/80 heterodimer. Subsequently, the DNA-dependent protein kinase catalytic subunit (DNA-PKcs), which is a member of the phosphatidylinositide 3-kinase-related kinases (PIKK), interacts with the Ku70/80 complex. At this stage, various end-processing factors are recruited for modifying the DNA ends which can include APE1, polynucleotide kinase/phosphatase (PNKP), Aprataxin, Aprataxin-and-polynucleotide kinase (PNK)-like factor (APLF), tyrosyl-DNA phosphodiesterase 1 and 2 (TDP1 and TDP2) and Artemis (Refs 33, 34). Finally, the complex of X-ray cross-complementing protein 4 with DNA ligase IV (XRCC4-Lig IV) completes the ligation stage of C-NHEJ. During A-NHEJ, and in contrast to C-NHEJ that utilises Ku70/80, PARP1 binds to the DSB ends and then mediates poly(ADP ribosyl)ation to act as a scaffold to recruit other proteins, such as MRE11/RAD50/NBS1 (MRN), PNKP, Artemis and Lig III-XRCC1 (Refs 35–37). The MRN complex and CtIP (carboxy-terminal binding protein-interacting protein) initiates the resection of DNA ends and generation of regions of micro-homology (Ref. 38) and finally Lig III-XRCC1 completes the A-NHEJ pathway via DNA end ligation. Interestingly, studies have revealed the possibility that A-NHEJ can be finalised in a Lig III- or Lig I-dependent process (Ref. 39).

### Homologous recombination (HR)

The HR pathway of DSB repair is predominantly initiated at the S/G_2_ phases of the cell cycle, and is an error-free mechanism that utilises homologous sister chromatids for repair (Refs 29, 40). A number of studies have identified that there are at least three sub-pathways of HR, including break-induced repair, synthesis-dependent strand annealing and classical HR. However commonly these pathways involve recruitment of the MRN complex that initiates the generation of short 3′-DNA single-stranded regions (Refs 41, 42) in a breast cancer protein 1 (BRCA1)-dependent manner, which are subsequently stabilised by replication protein A (RPA). BRCA2 is recruited which binds to the RAD51 DNA recombinase and locates it to the resected DNA ends displacing RPA. RAD51 bound to single-stranded DNA forms a nucleoprotein filament that searches for homology in the sister chromatid through strand invasion (Refs 43–46) (Fig. 3). Following DNA synthesis and branch migration, this yields Holliday junctions that are acted on by resolvases either in the same or opposite orientation (Ref. 47).

## Mechanisms contributing to radiosensitivity of HPV-positive and HPV-negative HNSCC

Given the established improved response of HPV-positive oropharyngeal HNSCC to radiotherapy than the corresponding HPV-negative cancers, cellular mechanisms and pathways have been investigated to provide details on those contributing to the differential radiosensitivity. In general, these studies have been conducted in immortal tumour cell lines, and utilising clonogenic assays (unless otherwise indicated below) as a gold standard for assessment of radiosensitivity. This has subsequently led to identification of key cellular pathways that modulate HNSCC radiosensitivity, not just in the context of HPV status, but also HNSCC overall (Table 1).

**Table 1.** Cellular pathways contributing to the radiosensitivity of HNSCC cells

Cellular pathways	Cell line(s)	Comment	Reference
AKT	SQ20B (HPV-negative)	Inhibition of AKT phosphorylation by gefitinib- or LYS294002-enhanced radiosensitivity	49
AKT	SQ20B (HPV-negative), UPCI-SCC90 and UMSCC47 (HPV-positive)	SQ20B cells with increased AKT phosphorylation were more radioresistant. All cells radiosensitised by treatment with nelfinavir	53
SMG-1	SCC25, SCC61 and JHU012 (HPV-negative), UPCI-SCC90 and UMSCC47 (HPV-positive)	Lower expression of SMG-1 in radiosensitive HPV-positive cells. Increased radiosensitivity of SCC61 and JHU012 cells following *smg-1* shRNA	56
β_1_ Integrin	Cal33, FaDu, HSC4, SAS, UTSCC5, UTSCC8, UTSCC14, UTSCC15, UTSCC45, XF354 (HPV-negative)	Eight out of 10 cell lines (not FaDu or SAS) radiosensitised in the presence of β_1_ integrin monoclonal antibody. UTSCC15 and XF354 radiosensitised following β_1_ integrin siRNA	57
β_1_ Integrin	UTSCC15, UTSCC45 and Cal33 (HPV-negative)	β_1_ integrin monoclonal antibody enhanced radiosensitivity of UTSCC15, UTSCC45 and Cal33 cells	58
DSB repair	Cal33, HSC4, FaDu, SAT, UTSCC5 (HPV-negative); UTSCC45, 93-VU147T, UDSCC2, UMSCC47, UPCI-SCC154 (HPV-positive)	HPV-positive cells had high residual levels of DSBs 24 h post-IR, which negatively correlated with cell survival	60
DSB repair	UMSCC1 (HPV-negative); UPCI-SCC154 and UMSCC47 (HPV-positive)	HPV-positive cells had high residual levels of DSBs ~4–8 h post-IR, and displayed reduced protein levels of DNA-Pk and BRCA2	61
DSB repair	UMSCC6 and UMSCC74A (HPV-negative); UMSCC47 and UPCI-SCC090 (HPV-positive)	HPV-positive cells had high residual levels of DSBs ~2–4 h post-IR, and UPCI-SCC090 cells displayed significantly reduced protein levels of Ku80, DNA-Pk, 53BP1 and BRCA2	64
DSB repair	SQD9 (HPV-negative); UPCI-SCC90 and UPCI-SCC154 (HPV-positive)	HPV-positive cells displayed reduced RAD51 foci post-IR	63
SSB repair	UMSCC6 and UMSCC74A (HPV-negative); UMSCC47 and UPCI-SCC090 (HPV-positive)	HPV-positive cells had higher capacity for SSB repair post-IR, and displayed significantly higher protein levels of XRCC1, Pol β, PNKP and PARP-1	64

### AKT signalling pathway increases radioresistance of HNSCC cells

Activation of overexpressed oncogenes (e.g. RAS and epidermal growth factor receptor (EGFR)) and loss of tumour suppressor genes (e.g. PTEN) leads to constitutive activation of the phosphatidyl inositol 3-kinases (PI3K)/AKT pathway that can enhance radioresistance of a various number of cancers (Ref. 48). In HNSCC tumour samples from an Indian population, it has been demonstrated that there is increased expression of activated, phosphorylated AKT (Refs 49, 50) and that this is as a consequence of loss of PTEN expression (Ref. 51). Loss of PTEN expression though, is not a widely observed phenomenon in HNSCC (Ref. 52). Regarding the specific association of AKT signalling with the radiobiology of HNSCC, inhibition of AKT phosphorylation in SQ20B HPV-negative HNSCC cells by either Iressa (gefitinib; 1 μM) or LYS294002 (10 μM) was shown to increase the sensitivity of the cells to IR (Ref. 49). This study was supported by observations that the relatively radioresistant HPV-negative HNSCC cell line SQ20B, compared with the more radiosensitive HPV-positive HNSCC cells (UPCI-SCC90 and UMSCC47), had increased activation of EGFR and consequently signalling through AKT phosphorylation at threonine 308 (Ref. 53). The radiosensitivity of all three cell lines was found to increase following incubation with the human immunodeficiency virus (HIV) protease inhibitor, nelfinavir (10 μM). In relation to the events through which AKT activation can promote radioresistance, independent studies have suggested that this is mediated through altered efficiency of IR-induced DNA DSB repair, albeit not specifically in HNSCC cells. AKT through the glycogen synthase kinases-3 beta (GSK3β)/β-catenin/lymphoid enhancer binding factor 1 (LEF-1) pathway can lead to upregulation of the MRE11 component of the MRN complex, and that MRE11-dependent phosphorylation of AKT can promote DSB repair and cell survival post-IR (Refs 54, 55).

### Downregulation of SMG-1 enhances radiosensitivity of HNSCC

E6 and E7 oncoproteins have been shown to downregulate suppressor with morphogenetic effect on genitalia (SMG-1) protein via hypermethylation of the promoter region of the gene. By immunohistochemistry analysis of forty HNSCC tissues (23 HPV-positive and 17 HPV-negative), the majority of HPV-positive tissues had negative or weak SMG-1 staining, although this was not statistically different compared with the HPV-negative tissues (Ref. 56). Quantitative polymerase chain reaction analysis also demonstrated that *smg-1* gene expression was generally lower in HPV-positive tumours, but again the data were not significant. This observation was supported by visibly lower SMG-1 protein levels in two HPV-positive HNSCC cell lines (UMSCC47 and UPCI-SCC90) versus three HPV-negative HNSCC cell lines (SCC25, SCC61 and JHU012), but also that transient expression of E6/E7 in HPV-negative cells (SCC61) appeared to decrease SMG-1 expression at both the mRNA and protein levels. A direct role for SMG-1 in controlling radiosensitivity of HNSCC cells was provided by shRNA knockdown of *smg-1* in two HPV-negative HNSCC cell lines (SCC61 and JHU012), where there was an observed reduced clonogenic survival following IR. Although this study suggested that levels of SMG-1 protein are negatively correlated with HPV infection and therefore impact on sensitivity of HPV-positive HNSCC cells to IR, further studies to support this have not been forthcoming.

### Elevated radiosensitivity by inhibition of β_1_ integrin

β_1_ integrin is a transmembrane cell adhesion receptor and mediates interaction to the extracellular matrix to promote cell survival. It has been demonstrated that inhibition of β_1_ integrin using a monoclonal antibody (10–100 μg/ml) enhanced the radiosensitivity of eight out of a panel of 10 HPV-negative HNSCC cell lines (Table 1), and which was supported by increased radiosensitivity of two of these cell lines (UTSCC15 and XF354) following β_1_ integrin siRNA (Ref. 57). The growth of UTSCC15-derived xenografts in immunocompromised nude mice was also significantly delayed in the presence of β_1_ integrin inhibition. The mechanism of radiosensitisation through targeting β_1_ integrin was shown to be mediated through focal adhesion kinase (FAK) and ultimately in downregulation of c-jun NH_2_-terminal kinase (JNK) signalling after IR induction. A study from the same research group demonstrated that β_1_ integrin monoclonal antibodies (10 μg/ml) in HPV-negative HNSCC cells (UTSCC15, UTSCC45 and Cal33) led to a higher level of unrepaired DSBs post-irradiation as analysed by γH2AX/53BP1 foci and comet assay analysis, and increased γH2AX/53BP1 foci was also confirmed in UTSCC15-derived xenografts (Ref. 58). Of note here is that although UTSCC45 cells are classed as HPV-negative, they have been shown to contain HPV type-33 (Ref. 59). The importance of FAK and JNK signalling in this mechanism was confirmed using siRNA experiments in UTSCC15 and Cal33 cells that recapitulated the increased residual levels of DSBs post-IR. Furthermore, using NHEJ reporter assays decreased efficiency of NHEJ was demonstrated and there appeared to be significantly reduced protein expression of Ku70, particularly in UTSCC15 and Cal33 cell lines, following β_1_ integrin inhibition suggesting a specific impact on C-NHEJ contributing to DSB persistence. Interestingly the combined inhibition of β_1_ integrin and PARP (via olaparib) further radiosensitised HNSCC cells (UTSCC15, UTSCC45 and Cal33), suggesting synergy between the inhibition of NHEJ and BER. However further progress and studies clarifying the role of β_1_ integrin in regulating HNSCC radiosensitivity, and any potential dependence on HPV status are lacking.

### HPV-positive HNSCC displays impaired DNA DSB repair

Given that IR principally targets DNA to generate its cell killings effects, it is understandable that the major focus of some studies has been to correlate differences in radiosensitivity of HPV-positive versus HPV-negative HNSCC to efficiencies of DNA damage repair. Cumulatively, these studies have revealed a strong downregulation of DSB repair capacity in HPV-positive HNSCC, which appears to be the major driver contributing to increased radiosensitivity, although there are a few reported discrepancies in the precise mechanism contributing to this phenotype. By comparing five HPV-positive and five HPV-negative HNSCC cell lines (Table 1), although it was demonstrated that there was a high variability in radiosensitivity, in general the HPV-positive cells were more radiosensitive and that the three most sensitive (UPCI-SCC154, UMSCC47 and UDSCC2) showed high residual levels of DSBs 24 h post-IR via detection of γH2AX/53BP1 foci (Ref. 60). This was also associated with a sustained G_2_-arrest in the HPV-positive HNSCC cells, indicative of a prolonged activation of the G_2_M checkpoint. A subsequent study also demonstrated that two HPV-positive HNSCC cell lines (UMSCC47 and UPCI-SCC154) harbour defects in DSB repair, as shown via the persistence of γH2AX foci at 8–12 h post-IR versus HPV-negative HNSCC cells (UMSCC1). Direct visualisation of increased DSB levels was demonstrated only in UMSCC47 cells 4 h post-IR, as shown via neutral comet assays, although no statistical analysis on these data was performed (Ref. 61). However, the study was progressed further by showing that the HPV-positive HNSCC cells had defects in both NHEJ and HR, through the failure to generate DNA-Pk and BRCA2/RAD51 foci, respectively in response to IR. In fact, reduced protein levels of DNA-Pk and BRCA2, and to some extent RAD51, were observed in these cells compared with the UMSCC1 HPV-negative counterparts, which are reported to be p53-deficient (Ref. 62). Impairment of HR in two HPV-positive cell lines (UPCI-SCC090 and UPCI-SCC154) versus one HPV-negative cell line (SQD9) through decreased RAD51 foci formation at 4 h post-irradiation, has also been previously observed, mediated by high levels of p16INK4a (Ref. 63).

Data generated from our lab have corroborated that the increased radiosensitivity of two HPV-positive HNSCC cell lines (UMSCC47 and UPCI-SCC90) versus two HPV-negative HNSCC cell lines (UMSCC6 and UMSCC74A) is via defective DSB repair, as revealed through the persistence of DSBs at least 2–4 h post-IR by the neutral comet assay (Ref. 64). There was also a significant persistence of γH2AX and 53BP1 foci (in UMSCC47 cells) at 4–8 h following irradiation indicative of a NHEJ defect, but in contrast to the previous study described above, these cells still appeared to develop RAD51 foci. Additionally, via quantitative immunoblotting analysis, only one of the HPV-positive HNSCC cell lines (UPCI-SCC90) demonstrated significantly reduced levels of proteins involved in NHEJ and HR (Ku86, DNA-Pk, 53BP1 and BRCA2). The discrepancies may relate to the fact that the previous study utilised only one HPV-negative UMSCC1 cell line, which as mentioned above lacks p53 expression, whereas our study used HPV-negative HNSCC cell lines that all expressed wild-type p53. Therefore, although our study would suggest a variability in the precise mechanism through which defective DSB repair is attained in HPV-positive cells, this would nevertheless support that a deficiency in NHEJ plays a major role in this phenotype. Indeed, recent evidence has shown that HPV-positive HNSCC do not demonstrate hallmarks of HR deficiency, and that at least in U2OS cells that the overexpression of the HPV E7 oncoprotein leads to downregulation of NHEJ activity, as observed by the lack of phosphorylated DNA-Pk foci formation post-irradiation (Ref. 65). Interestingly in our study, we also described a significant upregulation of SSB repair proteins in HPV-positive HNSCC cells (both UMSCC47 and UPCI-SCC90), including XRCC1, Pol β, PARP-1 and PNKP, in comparison with HPV-negative HNSCC cell lines (UMSCC6 and UMSCC74A) (Ref. 64). This equated to an increased capacity for SSB repair in HPV-positive HNSCC cells, as visualised by alkaline comet assays. Although this is not contributory to the increased cellular radiosensitivity, it does suggest that these cells upregulate BER as a consequence of defective DSB repair, and that HPV infection has a more dramatic global impact on repair protein expression and capacity for DNA damage repair. This work should be advanced further using a greater comparative number of HPV-positive and HPV-negative cells, including primary cells derived from HNSCC patients.

## Strategies for radiosensitisation of HNSCC

The evidence described above has provided indications as to the key mechanisms and cellular pathways that contribute to the radiosensitivity of HNSCC, particularly comparing HPV-positive with relatively radioresistant HPV-negative HNSCC. Based on these observations, this has provided specific cellular targets for drugs and small molecule inhibitors that can exacerbate the cell killing effects of radiotherapy, and ultimately in improving HNSCC cancer treatment.

### AKT inhibition

The HIV protease inhibitors have been suggested to be effective radiosensitisers in a number of different cancers because of their AKT inhibition action (Ref. 48). Specifically relating to HNSCC, two inhibitors (amprenavir, 10 μM; and nelfinavir, 5 μM) shown to inhibit AKT phosphorylation were demonstrated to increase the radiosensitivity of SQ20B HPV-negative HNSCC cell lines, which harbour an EGFR mutation (Ref. 66). Amprenavir and nelfinavir also acted synergistically in combination with IR in supressing growth of SQ20B-derived xenografts. The radiosensitisation of SQ20B cells by nelfinavir was confirmed in two further studies (Refs 53, 67). One of these studies demonstrated that nelfinavir furthermore decreased survival of two HPV-positive HNSCC cells (UMSCC47 and UPCI-SCC090) post-IR, although reported that the mechanism of radiosensitisation for these cells was mediated via phosphatase and tensin homologue (PTEN), whereas SQ20B cells was caused through EGRF hyperactivation (Ref. 53).

### PARP inhibition

Given that there is accumulating evidence that HPV-positive HNSCC cells harbour defects in DSB repair efficiency, it is understandable that inhibition of PARP that coordinates BER has been investigated as a mechanism to further radiosensitise cells through synthetic lethality, in a similar context to BRCA-deficient tumours (Refs 68, 69). However, these studies have revealed some interesting results, particularly with PARP inhibition in HPV-negative HNSCC cells (Table 2). Note that the ability of PARP inhibitors to cause radiosensitisation may relate to inhibition of PARP activity, trapping of PARP on the DNA, or a combination of both. As such, the majority of the studies in HNSCC cells have utilised olaparib which is considered a strong inhibitor and PARP-trapper, whereas veliparib is a relatively weak PARP-trapping agent (Ref. 70). Consistent with the above hypothesis, the PARP inhibitor olaparib (1 μM) was shown to radiosensitise five HPV-positive HNSCC cell lines (UTSCC45, 93-VU147T, UDSCC2, UMSCC47 and UPCI-SCC154) (Ref. 71). This is supported by data demonstrating that the radiosensitivity of a HPV-positive HNSCC cell line (UPCI-SCC154) was significantly enhanced in combination with a relatively high dose of veliparib (10 μM), in comparison with a HPV-negative HNSCC cell line (UMSCC1), which was mediated through increases in γH2AX foci and ultimately apoptosis (Ref. 61). However, the increased radiosensitivity of another HPV-positive HNSCC cell line (UMSCC47) versus the vehicle control was not apparent. Nevertheless, this study also demonstrated that veliparib alone was able to cause tumour growth delay of HPV-positive xenografts (UMSCC47 and one patient-derived) in immunocompromised mice. Veliparib at high doses (10 and 20 μM) has additionally been described to enhance the radiosensitivity of three HPV-positive HNSCC cells (UMSCC47, UPCI-SCC104 and UPCI-SCC154), but also one of three HPV-negative HNSCC cells (SQD9 versus SC263 and Cal27) (Ref. 72). However, this study utilised xenografts to show that addition of veliparib in combination with IR had a significant, but only mild impact on growth of HPV-positive UPCI-SCC154-derived tumours, but that the lack of growth of HPV-negative SD9-derived tumours post-irradiation did not allow for comparative effects. Work from our lab has also shown that the sensitivity of the UMSCC47 HPV-positive HNSCC cell line could be enhanced with olaparib at a much lower concentration (0.1 μM), although we did not see any impact of the PARP inhibitor on the radiosensitivity of another HPV-positive HNSCC cell line (UPCI-SCC090) (Ref. 64).

**Table 2.** Comparison of studies demonstrating radiosensitisation of HNSCC cells in combination with PARP inhibition

PARP inhibitor	Cell line(s)	Comment	Reference
Veliparib	UMSCC47 and UPCI-SCC154 (HPV-positive); UMSCC1 (HPV-negative)	10 μM inhibitor used, increased radiosensitisation of UPCI-SCC154 cells	61
Veliparib	SQD9, SC263 and Cal27 (HPV-negative); UMSCC47, UPCI-SCC104 and UPCI-SCC154 (HPV-positive)	10 and 20 μM inhibitor used, increased radiosensitisation of all HPV-positive cells, and SQD9 cells; mildly delayed growth of UMSCC47-derived xenografts	72
Olaparib	UTSCC45, 93-VU147T, UDSCC2, UMSCC47, UPCI-SCC154 (all HPV-positive)	1 μM inhibitor used, further enhanced radiosensitisation in 93-VU147T and UMSCC47 in combination with CHK1 inhibition	71
Olaparib	UMSCC47 and UPCI-SCC090 (HPV-positive); UMSCC74A and UMSCC6 (HPV-negative)	0.1 μM inhibitor used, increased radiosensitisation of HPV-negative cells	64
Olaparib	Cal33, UTSCC15 and UTSCC45 (HPV-negative)	1 μM inhibitor used, further enhanced radiosensitisation in combination with β_1_ integrin inhibition	58
Olaparib	Cal33, FaDu, HSC4, SAS, UTSCC5, UTSCC8, UTSCC14, UTSCC15, UTSCC45 and XF345 (HPV-negative)	1 μM inhibitor used, radiosensitisation more effective in cells with HR-deficiency	73

Intriguingly, there is evidence suggesting that HPV-negative HNSCC cells also display elevated sensitivity to IR in the presence of PARP inhibitors, despite being DSB repair proficient. Olaparib treatment (1 μM) of HPV-negative HNSCC cells (UTSCC15, UTSCC45 and Cal33) was observed to cause enhanced radiosensitisation through accumulation of DSBs, as revealed by increases in γH2AX/53BP1 foci 24 h post-irradiation (Ref. 58). This study also demonstrated a synergistic effect of inhibition of β_1_ integrin with olaparib in exacerbating the cell killing effects of IR in these HPV-negative HNSCC cells. Data obtained from our lab have also shown that olaparib (0.1 μM) significantly increased the radiosensitivity of two HPV-negative HNSCC cells (UMSCC6 and UMSCC74A), which was in fact more pronounced than the impact on two HPV-positive HNSCC cell lines (UMSCC47 and UPCI-SCC090) as revealed by higher dose enhancement ratios (Ref. 64). It has been suggested that the radiosensitisation of HNSCC cells in the presence of PARP inhibition is very much dependent on the efficiency of HR. Using a panel of HPV-negative HNSCC cells (Table 2) and measurement of HR capacity through reporter assays, cells deficient in HR were demonstrated to be generally more effectively radiosensitised in combination with olaparib (1 μM), than those that were HR-proficient (Ref. 73). Finally, a recent study has further revealed the variability in the response of both HPV-positive and HPV-negative HNSCC cells (four of each) to olaparib, although only as a monotherapy (Ref. 74). Only two HPV-positive cell lines (UPCI-SCC090 and CUOP2) were deemed sensitive to olaparib (0.5–1 μM) and one HPV-negative HNSCC cell line (UMSCC74A) demonstrated intermediate sensitivity. However, unfortunately no data were presented on combination of olaparib with IR.

Taken together, although there is evidence demonstrating that HPV-positive HNSCC are radiosensitised by PARP inhibition which is consistent with these harbouring DSB repair defects, further evidence supports that HPV-negative HNSCC can also be radiosensitised under these conditions. However, the specific mechanism through which this is achieved, and whether this is dependent on NHEJ/HR capacity or related to the cellular levels of PARP proteins that the inhibitors target, is currently unclear.

### DSB repair inhibition

The protein kinases DNA-Pk, ATM and ATR play critical roles in the signalling and coordination of repair of DSBs. A few studies have reported their targeting in HNSCC cells to increase radiosensitivity, with most of these directed towards ATR (Table 3). One study demonstrated that siRNA knockdown of DNA-Pk significantly decreased the survival of two HPV-negative HNSCC cell lines (UTSCC15 and UTSCC45) post-irradiation, because of the persistence of DSBs as revealed by γH2AX/53BP1 foci increases at 24 h post-irradiation (Ref. 58). No synergistic effect of DNA-Pk knockdown in the presence of β_1_ integrin inhibition on cellular radiosensitivity was seen. Targeting DNA-Pk activity to enhance radiosensitisation was shown in another study using a specific inhibitor (KU0060648, 0.25 μM) in HPV-negative HNSCC cells (HN4 and HN5) (Ref. 75). Very recently, a DNA-Pk inhibitor (IC87361, 3.3 μM) was demonstrated to enhance the radiosensitivity of three HPV-negative HNSCC cell lines (UTSCC54C, UTSCC74B and UTSCC76B) by clonogenic assays, with an indication that this approach is more effective in these cells under the specific assay conditions (cells plated 24 h post-IR) than the combination of IR with PARP inhibition by olaparib (Ref. 76). Furthermore, another recent study showed that the DNA-Pk inhibitor NU7441 (1 and 2.5 μM), was effective in enhancing radiosensitisation of both HPV-negative (SQD9, SC263 and Cal27) and HPV-positive (UMSCC47, UPCI-SCC104 and UPCI-SCC154) HNSCC cell lines (Ref. 72). The persistence of γH2AX foci 24 post-IR, and therefore of DSBs, in combination with DNA-Pk inhibition was demonstrated in two of the cell lines (SQD9 and UPCI-SCC154). This study was extended further using in vivo models, which provided evidence that treatment of HPV-negative (SQD9) and HPV-positive (UPCI-SCC154) HNSCC xenografts, but also HPV-negative HNSCC patient-derived xenografts (HNC019 and HNC021), with NU7441 in combination with IR led to delayed tumour growth. This is supported by our recent data, showing significantly enhanced radiosensitisation of two HPV-negative (UMSCC6 and UMSCC74A) and one of two HPV-positive (UMSCC47 but not UPCI-SCC090) HNSCC cells and 3D spheroids in the presence of a DNA-Pk inhibitor (KU57788, 1 µM), following both X-rays and protons (Ref. 77). The impact of KU57788 in combination with IR in reducing 3D spheroid growth of additional HPV-negative (FaDu and A253) HNSCC cells was also shown.

**Table 3.** Comparison of studies demonstrating radiosensitisation of HNSCC cells in combination with DSB repair inhibition

Inhibition strategy	Cell line(s)^a^	Comment	Reference
DNA-Pk siRNA	UTSCC15 and UTSCC45	No synergy of radiosensitisation with β_1_ integrin inhibition	58
DNA-Pk inhibitor (KU0060648)	HN4 and HN5	0.25 μM inhibitor used, further enhanced radiosensitisation in combination with ATR inhibition (AZD6738)	75
DNA-Pk inhibitor (IC87361)	UTSCC54, UTSCC74B and UTSCC76B	3.3 μM inhibitor used	76
DNA-Pk inhibitor (NU7441)	SQD9, SC263 and Cal27; UMSCC47, UPCI-SCC104 and UPCI-SCC154 (HPV-positive)	1 and 2.5 μM inhibitor used, enhanced radiosensitisation of all cells; also delay in growth of SQD9 and UMSCC47-derived xenografts	72
ATM inhibitor (GSK635416A)	UTSCC2, UTSCC8, UTSCC24A, UTSCC36 and UTSCC40	2 μM inhibitor used, further enhanced radiosensitisation of UTSCC36 and UTSCC40 in combination with PARP inhibition (olaparib)	78
ATR siRNA	UPCI-SCC029B, UPCI-SCC040 and UPCI-SCC131		79
ATR inhibitor (VE821)	SQ20B	1 μM inhibitor used	80
ATR inhibitor (AZD6738)	Cal27, FaDu,	0.25 μM inhibitor used	81
ATR inhibitor (AZD6738)	HN4 and HN5	0.25 μM inhibitor used, further enhanced radiosensitisation in combination with DNA-Pk inhibition (KU0060648)	75
ATM (KU55933); ATR (VE821) and DNA-Pk (KU57788) inhibitors	UMSCC6 and UMSCC74A; UMSCC47 and UPCI-SCC090 (HPV-positive)	10 μM (ATM) and 1 μM (ATR and DNA-Pk) inhibitors used, enhanced radiosensitisation of all cells following photons and protons apart largely from UPCI-SCC090.	77

a The majority of cell lines employed were HPV-negative, unless stated.

Utilising a small molecule inhibitor screen incorporating cell viability as an end-point in HNSCC cells, the compound GSK635416A was demonstrated to enhance the radiosensitivity of three HPV-negative HNSCC cell lines (UTSCC24A, UTSCC36 and UTSCC40) at a fixed IR dose of 4 Gy, because of the inhibition of ATM kinase activity and subsequent accumulation and persistence of DNA DSBs, as revealed by gel electrophoresis (Ref. 78). Additionally, the combination of GSK635416A (2 μM) with the PARP inhibitor olaparib (up to 10 μM), led to further enhanced radiosensitisation of UTSCC36 and UTSCC40 cells demonstrating additive effects of targeting both ATM and PARP. Significant radiosensitisation of an additional two HPV-negative HNSCC cells (UTSCC2 and UTSCC8) with GSK635416A, and synergy with olaparib, was also demonstrated in this study. Recent data from our lab has demonstrated enhanced radiosensitisation of two HPV-negative (UMSCC6 and UMSCC74A) and one HPV-positive (UMSCC47) HNSCC cells to both X-rays and protons in the presence of an ATM inhibitor (KU-55933, 10 µM) (Ref. 77). The inhibitor was also effective in supressing growth of 3D spheroids of HPV-negative (UMSCC74A, FaDu and A253) HNSCC cells in combination with IR, but had no impact on radiosensitivity of HPV-positive (UPCI-SCC090) HNSCC cells and 3D spheroids that are the most radiosensitive.

An siRNA knockdown of ATR has been shown to significantly increase the radiosensitivity of three HPV-negative HNSCC cells (UPCI-SCC029B, UPCI-SCC040 and UPCI-SCC131) containing loss of chromosome 11q, which is associated with increased radioresistance and poor patient prognosis (Ref. 79). In a limited study, the ATR inhibitor VE821 (1 μM) was demonstrated to enhance the radiosensitivity of HPV-negative HNSCC cells (SQ20B) (Ref. 80). This is supported by our recent evidence that the presence of VE821 (1 μM) can increase radiosensitisation of two HPV-negative (UMSCC6 and UMSCC74A) and one HPV-positive (UMSCC47) HNSCC cells and 3D spheroids in response to both X-rays and protons (Ref. 77). Utilisation of an alternative ATR inhibitor (AZD6738, 0.25 μM) has been observed to enhance radiosensitivity of four HPV-negative HNSCC cells in two separate studies (Cal27 and FaDu (Ref. 81); HN4 and HN5 (Ref. 75)), mediated through abrogation of HR (via γH2AX/RAD51 foci) and of the cell cycle, with subsequent increased apoptosis. The former study also demonstrated that 3D spheroid growth of FaDu cells was severely impeded by the combination of AZD6738 (1 μM) with irradiation (20 Gy in 10 fractions). However, the latter study demonstrated the significant cell killing effects of the combination of inhibition of both ATR (AZD6738) and DNA-Pk (KU0060648) with irradiation. Cumulatively, these data suggest that particularly targeting HR through ATR inhibition is able to sensitise HPV-negative HNSCC cells to IR, but which requires further validation in multiple cell lines in vitro, as well as demonstrating the impact of this strategy in appropriate in vivo models.

### Cell-cycle checkpoint inhibitors

During the cell cycle, activation of either the G_1_/S or G_2_/M checkpoints by CHK1 or CHK2 (Fig. 1) ensure that the cell can undergo DNA damage repair prior to DNA replication and cell division. Therefore these, and other downstream signalling kinases (including the Wee1-like protein kinase; WEE1), have been investigated as targets for improving HNSCC radiosensitivity, particularly in HPV-negative HNSCC cells that should contain unperturbed cell-cycle checkpoint activation mechanisms (Table 4). One study demonstrated a reduction of cellular survival in HPV-negative HNSCC cells lacking distal chromosome 11q (UPCI-SCC040, UPCI-SCC029B and UPCI-SCC131) treated with CHK1 siRNA in combination with irradiation (Ref. 79). Additionally, the CHK1 inhibitor PF0477736 (0.54 μM inhibitor) was demonstrated to increase radiosensitivity of two of these cell lines (UPCI-SCC040 and UPCI-SCC131), but not in UPCI-SCC066 cells without loss of distal chromosome 11q. An alternative inhibitor of CHK1, SAR020106 (0.125 μM), significantly elevated the radiosensitivity of two HPV-negative HNSCC cells (Cal27 and HN6) through cell-cycle perturbation, but also caused a significant delay in the growth of Cal27-derived xenografts in combination with radiation (Ref. 82). More recently the CHK1 inhibitor CCT244747 (0.7 μM) was also found to enhance the radiosensitivity of two HPV-negative HNSCC cells (NH4 and HN5) because of abrogated cell-cycle arrest, but which could be further exacerbated in the presence of paclitaxel (Ref. 83). The effective combination of CCT244747 plus radiation, but more so the triple combination of CCT244747, paclitaxel and radiation, in the prevention of growth of HN5-derived xenografts was also shown in this study.

**Table 4.** Comparison of studies demonstrating radiosensitisation of HNSCC cells in combination with cell-cycle checkpoint inhibition

Inhibition strategy	Cell line(s)	Comment	Reference
CHK1 siRNA and CHK1 inhibitor (PF0477736)	UPCI-SCC029B, UPCI-SCC040, UPCI-SCC131 and UPCI-SCC066 (HPV-negative)	0.54 μM inhibitor used, no increased radiosensitisation of UPCI-SCC066 without loss of distal chromosome 11q	79
CHK1 inhibitor (SAR020106)	Cal27 and HN6 (HPV-negative)	0.125 μM inhibitor used, also delay in growth of Cal27-derived xenografts	82
CHK1 inhibitor (CCT244747)	NH4 and HN5 (HPV-negative)	0.7 μM inhibitor used, further enhanced radiosensitisation in combination with paclitaxel, also delay in growth of HN5-derived xenografts with triple therapy	83
CHK1 inhibitor (PF0477736)	UTSCC45, 93VU147T, UDSCC2, UMSCC47 and UPCI-SCC154 (HPV-positive), FaDu and Cal33 (HPV-negative)	0.15 μM inhibitor used, no increased radiosensitisation of UPCI-SCC154 or Cal33	84
CHK1 inhibitor (PF0477736)	93-VU147T and UMSCC47 (HPV-positive)	0.15 μM inhibitor used, only single radiation dose examined, further enhanced radiosensitisation in combination with PARP inhibition (olaparib)	71
CHK1 inhibitors (LY2603618 and MK8778)	UPCI-SCC154, UMSCC47 and UDSCC2 (HPV-positive)	0.24 μM LY2603618 and 0.48 μM MK8778 used, slight enhanced radiosensitisation in combination with WEE1 inhibition (AZD1775)	85
CHK1/2 inhibitor (prexasertib)	UMSCC1, UMSCC2, UMSCC6, and FaDu (HPV-negative), UMSCC47 and UPCI-SCC090 (HPV-positive),	1 or 10 nM inhibitor used, further enhanced radiosensitisation in combination with 0.5 μg/ml cetuximab, also delay in growth of UMSCC1 and UMSCC47-derived xenografts	86
WEE1 inhibitor (AZD1775)	UPCI-SCC154 (HPV-positive)	60 nM inhibitor used	85

Studies have also analysed the comparative effect of CHK1 inhibition on radiosensitisation of HPV-positive and HPV-negative HNSCC cells. Utilising the CHK1 inhibitor PF0477736, it was shown that four out of five HPV-positive HNSCC cell lines (UTSCC45, 93VU147T, UDSCC2 and UMSCC47; but not UPCI-SCC154) were sensitised in the presence of the inhibitor (0.15 μM) because of a reduction in G_2_M arrest (Ref. 84). Furthermore, one HPV-negative HNSCC cell line (FaDu) but not another (Cal33) also revealed radiosensitisation following CHK1 inhibition. These data were partially supported by another study demonstrating that PF00477736 (0.15 μM) radiosensitised two of the same HPV-positive HNSCC cell lines (93VU147T and UMSCC47), albeit only analysing the response at a single 6 Gy dose of radiation (Ref. 71). Utilising alternative CHK1 inhibitors, LY2603618 (0.24 μM) and MK8778 (0.48 μM), the same group observed that both compounds radiosensitised three HPV-positive HNSCC cell lines (UPCI-SCC154, UMSCC47 and UDSCC2) to varying degrees and dependent on whether cells were plated at low density for irradiation or delayed plating following irradiation of exponentially growing cells (Ref. 85). Additionally, in this study the WEE1 inhibitor AZD1775 (60 nM) was shown to only largely enhance the radiosensitivity of one of the HPV-positive HNSCC cell lines (UPCI-SCC154), and the combination of AZD1775 with the CHK1 inhibitor (30 nM) appeared to marginally improve radiosensitivity of all three HPV-positive cell lines examined (Ref. 85). Finally, the CHK1/2 inhibitor prexasertib (1 or 10 nM dependent on cell line) exhibited similar effect on radiosensitisation, as analysed by cell proliferation, of four HPV-negative (UMSCC1, UMSCC2, UMSCC6 and FaDu) and two HPV-positive (UMSCC47 and UPCI-SCC090) HNSCC cell lines because of enhanced apoptosis (Ref. 86). Inhibition of cell proliferation was further exacerbated in the presence of cetuximab (0.5 μg/ml), and the impact of these combinatorial treatments were reproduced in vivo utilising UMSCC1- and UMSCC47-derived xenografts, which demonstrated the effective combination of prexasertib and IR, but more so the triple combination of prexasertib, cetuximab and IR, in supressing tumour growth. Given that there are observed variabilities in the radiation response of HNSCC cells to CHK1 inhibition, and the contribution of HPV status to this radiosensitivity, further preclinical research needs to be taken forward.

## Concluding remarks

The combination of targeting proteins involved in DNA damage repair (particularly PARP, DNA-Pk and ATR) and in cell-cycle checkpoint activation (particularly CHK1), have been demonstrated to enhance the radiosensitivity of HNSCC cells in vitro with some evidence also being generated in xenograft models in vivo. However, these studies have demonstrated the variability in the response dependent on the specific cell line and model utilised, and there are also discrepancies as to whether these strategies are selective for HPV-positive and/or HPV-negative HNSCC particularly because of the inherent and differential radiosensitivity of the cells through altered proficiency of DSB repair mechanisms. Nevertheless in general, evidence has been accumulating that these targeted strategies in combination with radiotherapy have the potential for further translation to the clinic for more effective treatment of HNSCC patients. Indeed, clinical trials utilising these combinational therapies are already underway (Ref. 87). In order for this to gather further momentum, it is clear that additional preclinical studies need to conducted not only a larger cohort of HNSCC cell lines, but also utilising 3D models including spheroids and patient-derived organoids, that more accurately reflect the structure and environment of the original tumour. This research should then be progressed further to in vivo models, such as the utilisation of multiple HNSCC-derived xenografts, to gain further evidence of the effectiveness of inhibitors targeting DNA damage repair or cell-cycle checkpoint activation in combination with IR in supressing HNSCC tumour growth. Accumulation of these data would then provide the solid basis for the future appropriate HNSCC clinical trials. However, the recent failure of two clinical trials in HPV-positive HNSCC targeting EGRF in combination with radiotherapy (discussed in Ref. 88), demonstrate the need for extensive preclinical studies to performed in a variety of cell lines and HNSCC tumour models before any targeted strategies are tested clinically.
